# Skeletal Health in Patients With Mitochondrial Diabetes: Case Series and Review of Literature

**DOI:** 10.1002/jbm4.10824

**Published:** 2023-09-26

**Authors:** Kagan Ege Karakus, Varun Suryadevara, Austin Larson, Prathosh Gangadhar, Viral N Shah

**Affiliations:** ^1^ Koç University, School of Medicine Istanbul Turkey; ^2^ Department of Endocrinology Jawaharlal Institute of Postgraduate Medical Education and Research (JIPMER) Puducherry India; ^3^ Department of Pediatrics, Section of Genetics University of Colorado Anschutz Medical Campus Aurora CO USA; ^4^ Department of Endocrinology IQRAA International Hospital and Research Centre Calicut India; ^5^ Barbara Davis Center for Diabetes University of Colorado Anschutz Medical Campus Aurora CO USA

**Keywords:** DIABETES, DXA, FRACTURE, MITOCHONDRIAL DIABETES, OSTEOPOROSIS, PRIMARY MITOCHONDRIAL DISEASE, SKELETAL HEALTH

## Abstract

Monogenic diabetes, including mitochondrial diabetes, constitutes 1% to 3% of all diabetes. Although there is an increased interest in understanding the mechanisms of bone fragility in people with diabetes, skeletal research is mostly focused on type 1 and type 2 diabetes. Little is known on skeletal health among people with mitochondrial diabetes. In this single‐center study, we presented clinical characteristics of individuals with mitochondrial diabetes and clinical diagnosis of osteoporosis. Of 10 patients with mitochondrial diabetes, 4 (40%) had a clinical diagnosis of osteoporosis. Patients with osteoporosis were older, had lower body mass index, longer diabetes duration, lower fasting C‐peptide, and presence of multiple comorbidities compared with patients without osteoporosis. In addition to our cases, we also systematically reviewed literature on skeletal health in people with mitochondrial diabetes and provided an overview of potential factors affecting skeletal health and future clinical and research directions to improve the care of people with mitochondrial disease. © 2023 The Authors. *JBMR Plus* published by Wiley Periodicals LLC on behalf of American Society for Bone and Mineral Research.

## Graphical abstract

Osteoporosis is common in people with mitochondrial diabetes. More research is needed to understand skeletal health in monogenic diabetes such as mitochondrial diabetes.

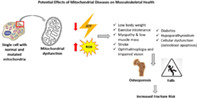

## Introduction

Skeletal health is adversely affected in the presence of diabetes. Fracture risk increases 4‐ to 6‐fold among people with type 1 diabetes (T1D) and 1.5‐ to 2‐fold among type 2 diabetes (T2D) compared with people without diabetes.^(^ ^1^ ^)^ Hyperglycemia, accumulation of advanced glycation end products (AGEs), and microvascular impairment are among a few factors implicated in the pathophysiology of bone fragility in T1D and T2D.^(^ ^2^ ^)^

Skeletal health is mostly studied in people with T1D and T2D, but little is known about skeletal health in other forms of diabetes, such as monogenic diabetes, which accounts for 1% to 3% of all diabetes.^(^ ^3^ ^)^ Primary mitochondrial diseases are defined as disorders affecting the structure or function of the mitochondria because of either nuclear DNA or mitochondrial DNA mutations.^(^ ^4^ ^)^ Diabetes is a common feature of many primary mitochondrial diseases.^(^ ^5^ ^)^ It is estimated that about 1% of T1D or T2D are mitochondrial diabetes but are unfortunately misclassified. The most common etiology of mitochondrial diabetes is the m.3243A > G pathogenic variant in the gene encoding the transfer RNA mt‐tRNA^Leu(UUR)^.^(^ ^5^ ^)^ Clinical presentations of the mitochondrial diseases are variable due to heteroplasmy; that is, there are variable numbers of mutated versus normal mitochondria within the organ.^(^ ^6^ ^)^ Besides diabetes, primary mitochondrial disorders also affect the musculoskeletal system and present with myopathy, scoliosis, and contractures, which predispose to fractures and poor bone health.^(^ ^4^ ^)^

In this case series, we aim to investigate (i) the prevalence of osteoporosis among people diagnosed with mitochondrial diabetes, and (ii) evaluate factors responsible for osteoporosis. We also reviewed literature on skeletal health among people with mitochondrial diabetes, which is summarized in this article.

## Methods

In this single‐center, cross‐sectional study, we systematically searched electronic medical records (EMR) to identify people with mitochondrial diabetes. Barbara Davis Center for Diabetes (BDC) is a clinical and research center that cares for an average of 1900 adults with diabetes (>95% T1D). The BDC caters to patients mostly from Colorado. This study was approved by Colorado Multiple Institutional Review under the exempt category.

Medical charts of identified patients with mitochondrial diabetes were manually reviewed for accuracy of diagnosis and the following information was collected: age, sex, duration of diagnosis, clinical presentation, complications and comorbidities related to diabetes and mitochondrial disease, glycated hemoglobin (HbA1c), anthropometric measures, and skeletal‐related outcomes such as any fractures, diagnosis of osteoporosis, and dual‐energy X‐ray absorptiometry (DXA) bone density if available. Clinical osteoporosis was defined based on physician's note in the EMR or International Classification of Diseases (ICD) codes indicative of osteoporosis or DXA bone mineral density (BMD). We also included a case of mitochondrial diabetes that was diagnosed and managed by one of the authors in India, who consulted with the BDC.

We searched PubMed on June 7, 2023, to identify literature on mitochondrial diabetes and skeletal health. First, we used terms “mitochondrial diabetes (tiab) AND fracture (tiab)” and did not find any articles. Then, we searched literature using the search term ([Mitochondrial diabetes OR MIDD OR maternally inherited diabetes] AND [fracture OR bone density OR Body composition OR DEXA or DXA]). Only human studies and published literature in English were included. Our initial search yielded 255 research publications. We also searched the Mitochondrial Medicine Society (http://www.mitosoc.org/) for their guidelines on management of mitochondrial disorders, and references of the guidelines were reviewed. Articles that reported fracture or bone density or bone structural properties were included in this review.

## Results

After the clinic EMR search and manual chart review, we identified nine people with genetically confirmed mitochondrial diabetes and one person with Wolfram syndrome. Though Wolfram syndrome is not typically considered a primary mitochondrial disease, we included this case as it is caused by a defect in calcium influx within the mitochondria leading to mitochondrial dysfunction.^(^ ^7^ ^)^

Of the 10 adult patients with mitochondrial diabetes, nine were female, the median (interquartile range [IQR]) age was 37.5 (23.75–41) years, and body mass index (BMI) was 22.9 (18.4–25.2) kg/m^2^. Nine patients were treated with insulin therapy, and one patient was receiving injectable once‐a‐week glucagon‐like peptide‐1 receptor agonist (GLP‐1RA) therapy. The median (IQR) of HbA1c was 7.9 (6.7–8.5)%. Four patients had the m.3243A > G pathogenic variant, three had the m.8363G > A pathogenic variant, one had the m.14709 T > C pathogenic variant, and one had a large deletion of mitochondrial DNA that resulted in Kearns Sayre Syndrome (KSS). Six patients had sensorineural hearing loss, five had muscle pain or weakness, three had migraines, two had abnormal renal function, two had hypothyroidism, two had external ophthalmoplegia, and two had cerebellar ataxia.

Of the 10 patients with mitochondrial diabetes, four patients had a clinical diagnosis of osteoporosis based on DXA BMD and/or history of fragility fractures (40%) and were receiving treatment for osteoporosis. Bisphosphonate was used in two patients, and an anabolic agent was used in another two patients. Characteristics of individuals with mitochondrial diabetes and osteoporosis are summarized in Table 1.

**Table 1:** Characteristics of Patients With Mitochondrial Diabetes and Osteoporosis

	Patient 1	Patient 2	Patient 3	Patient 4
Age (years)/sex	49/female	38/male	26/female	44/female
Race	Asian	Asian Indian	White	‐
Ethnicity	Non‐Hispanic	Non‐Hispanic	Hispanic	‐
Height/weight (cm/kg)	149.8/35.8	‐	144.8/35.8	154.9/44.8
BMI (kg/m^2^)	16	‐	17.1	18.4
Duration of diabetes (years)	29	10	1	12
Age at mitochondrial disease diagnosis (years)	45	38	17	40
HbA1c (%)^b^	6.7	11.5	7.9	7.1
Fasting C‐peptide (ng/mL)	0.63	0.37	0.41	1.5
Mitochondrial DNA variant (heteroplasmy if available)	m.3243A > G (91.3% in blood)	m.3243A > G	Large deletion of mitochondrial DNA	m.3243A > G (46% in buccal mucosa)
Sensorineural hearing loss	Yes	Yes	Yes	Yes
Other comorbidities	Stroke, renal transplant, muscle pain, hypothyroidism	Hypertrophic cardiomyopathy, hypertension, chronic kidney disease, fracture	Sick sinus syndrome, external ophthalmoplegia, muscle weakness, cerebellar ataxia, dysarthria	Severe endometriosis, migraines
DXA				
Left femoral neck BMD (g/cm^2^)	0.576	0.426 (gm/cm^3^)^a^	0.645	‐
*Z*‐score	−2	−3.1	−1.8	
*T*‐score	−3.3	−3.32		
Total hip BMD (g/cm^2^)	0.629	0.609 (gm/cm^3^)^a^	0.674	‐
*Z*‐score	−1.9	−2.53	−2.2	
*T*‐score	−3.3	−2.69		
Total lumbar spine BMD (g/cm^2^)	1.084	1.531 (gm/cm^3^)^a^	0.825	‐
*Z*‐score	0.3	−0.52	−2	
*T*‐score	−0.9	−0.88		

*Note*: – denotes missing data in the EMR.

Abbreviations: BMD = bone mineral density; BMI = body mass index; DXA = dual‐energy X‐ray absorptiometry; HbA1c = glycated hemoglobin.

^a^ Data based on quantitative CT scan of lumbar spine and hip.

^b^ HbA1c at the time of data collection.

Comparison of clinical characteristics of patients with clinical diagnosis of osteoporosis (*n* = 4) versus those without osteoporosis (*n* = 6) is presented in Table 2. Patients with osteoporosis were older, had a lower BMI, longer duration of diabetes, lower fasting c‐peptide, and a higher frequency of comorbidities than people without osteoporosis. The differences were statistically nonsignificant given the small sample size.

**Table 2:** Characteristics of Patients With Mitochondrial Diabetes by Osteoporosis

	Mitochondrial diabetes with osteoporosis (*n* = 4)^a^	Mitochondrial diabetes without osteoporosis (*n* = 6)^a^
Female/male, *n*	3/1	6/0
Age (years), median (IQR)	41 (29–47.8)	30 (22–47)
BMI (kg/m^2^), median (IQR)	17.1 (16–18.4)^b^	24.2 (22.5–27.5)
Diabetes duration (years), median (IQR)	11 (3.3–24.8)	7.5 (3.3–11.5)
HbA1c (%), mean ± SD	8.3 ± 2.2	7.7 ± 1.8
Fasting C‐peptide (ng/mL), median (IQR)	0.52 (0.38–1.28)	2.0 (0.4–2.5)^c^
% with sensorineural hearing loss	100%	33%
% with abnormal renal function	50%	17%

*Note*: Osteoporosis diagnosis was based on physician's note in the EMR or ICD codes indicative of osteoporosis or DXA BMD.

Abbreviations: BMD = bone mineral density; BMI = body mass index; DXA = dual‐energy X‐ray absorptiometry; HbA1c = glycated hemoglobin; IQR = interquartile range.

^a^ The differences in clinical characteristics were statistically nonsignificant.

^b^ BMI was missing for one patient.

^c^ Fasting C‐peptide levels were missing for three patients without osteoporosis.

Our systematic search included 255 publications. After our initial review, only four publications meeting our inclusion/exclusion criteria were included here (Supplemental Fig. S1). The summary of all four studies^(^ ^8^, ^9^, ^10^, ^11^ ^)^ with their key findings is provided in Table 3. In four studies, 55 of 151 patients with mitochondrial disorders had diabetes. In brief, the prevalence of osteopenia and/or osteoporosis was reported around 50%.^(^ ^9^, ^11^ ^)^ There was no significant difference noted in serum calcium or bone turnover markers in one study.^(^ ^9^ ^)^ Another study reported lower bone turnover markers in patients with mitochondrial disease compared with controls.^(^ ^8^ ^)^ However, when adjusted for diabetes, bone turnover makers were not significantly different between the two groups.^(^ ^8^ ^)^ Only one study reported bone structure using high‐resolution peripheral quantitative computed tomography (HR‐pQCT) and found that people with mitochondrial disease had a thinner cortex and lower volumetric BMD (vBMD) at the distal radius, leading to reduced bone strength.^(^ ^8^ ^)^ In almost all studies, BMI was reported to be lower in people with mitochondrial disease than controls without diabetes or controls with diabetes.^(^ ^8^, ^9^, ^10^, ^11^ ^)^ All studies were cross‐sectional, and we did not find any prospective study in the literature reporting skeletal outcomes in people with primary mitochondrial disease.

**Table 3:** Summary of Published Studies on Skeletal Health in People With Primary Mitochondrial Diseases

First author et al. (ref), year	Country	Study design	Sample size	% with diabetes	Male/female	Mean age (years)	Mean BMI	Key outcomes
Langdahl et al. 2017^(^ ^8^ ^)^	Denmark	Cross‐sectional, Case–control	45 adults with m.3243A > G 45 controls	55% cases 0% controls	16/29 cases 16/29 controls	47.6 cases 47.8 controls	21.4 cases 26.3 controls	LS, TH, and FN aBMD was lower in cases than controls. aBMD at FN was lower among those with DM than non‐DM. Bone turnover markers were significantly lower in cases than controls but confounded by diabetes status. The presence of m.3243A > G was significantly associated with lower total vBMD, cortical vBMD, and trabecular vBMD at radius by HR‐pQCT.
Zhu et al. 2017^(^ ^9^ ^)^	China	Cross‐sectional	9 MIDD 33 T1D 86 T2D	100%	4/5 MIDD 19/14 T1D 56/30 T2D	40.4 MIDD 39.2 T1D 41.8 T2D	18 MIDD 20.4 T1D 25.9 T2D	No significant differences in serum Ca, vitamin D3, PTH, bone resorption markers (CTX), and bone formation markers (OC) among three groups. Prevalence of osteoporosis was higher in MIDD group (50%) compared with T1D (21.8%) and T2D (11.1%).
Gandhi et al. 2017^(^ ^10^ ^)^	USA	Cross‐sectional, retrospective	80 primary mitochondrial disease	7.5%	33/47	5.7 at the time of fist assessment	NR	14% of the cohort had a prior history of fractures. Mean age at fracture was 6.4 years.
Geng et al. 2018^(^ ^11^ ^)^	China	Cross‐sectional	17 adults m.3243A > G 17 controls	88% cases 0% controls	9/8 cases 9/8 controls	51 cases 47 controls	NR	Osteopenia and/or osteoporosis was present in 53% of cases Higher heteroplasmy in leukocyte was associated with decreased level of total hip BMD *T*‐score even after adjustment for age, sex, and HbA1c.

Abbreviations: aBMD = areal bone mineral density; BMD = bone mineral density; BMI = body mass index; Ca = calcium; DM = diabetes mellitus; FN = femoral neck; HbA1c = glycated hemoglobin; HR‐pQCT = high‐resolution peripheral quantitative computed tomography; LS = lumbar spine; MIDD = mitochondrial diabetes; NR = not reported; PTH = parathyroid hormone; T1D = type 1 diabetes; T2D = type 2 diabetes; TH = total hip; vBMD = volumetric bone mineral density.

## Discussion

In our single‐center, cross‐sectional study, the prevalence of a clinical diagnosis of osteoporosis was 40%, similar to what was reported by two previous publications.^(^ ^9^, ^11^ ^)^ This may be underreporting considering most people with mitochondrial disease are not screened for osteoporosis. Osteoporosis prevalence in our cohort may be underreported as the diagnosis of osteoporosis was based on clinical diagnosis (ICD codes and/or DXA BMD), and it is possible that this prevalence could be higher if all of our patients with mitochondrial diabetes were offered DXA BMD for osteoporosis screening. In a large survey by the Mitochondrial Medicine Society involving 32 physicians running mitochondrial clinics in North America, osteoporosis screening and skeletal health preventive measures were rarely ordered or recommended,^(^ ^12^ ^)^ despite the fact that nearly 90% to 100% of patients with primary mitochondrial disease have some form of musculoskeletal complications or comorbidities.^(^ ^13^ ^)^

Body weight has a positive effect on bone density,^(^ ^14^ ^)^ and a lower BMI in people with mitochondrial disease may be one of the major factors affecting skeletal health among these people. Adequate nutrition and physical activity is recommended to promote a healthy weight in people with primary mitochondrial disorders.^(^ ^15^ ^)^

Diabetes alters bone health through multiple mechanisms such hyperglycemia‐induced bone matrix accumulation of AGEs, microvascular impairment, reduced osteoblast differentiation due to low insulin‐like growth factor 1, and reduced Wnt signaling.^(^ ^16^ ^)^ On the other hand, mitochondrial diseases cause mitochondrial dysfunction and impaired oxidative phosphorylation, leading to increased oxidative stress. The severity of mitochondrial diseases correlates with the oxidative stress. Moreover, presence of diabetes also depends on the severity of mitochondrial dysfunction.^(^ ^17^ ^)^ We believe that diabetes may not have a major independent effect on skeletal health in people with primary mitochondrial diseases. The direct and secondary effects of mitochondrial dysfunction (oxidative stress–induced osteoblastic dysfunction and apoptosis) may be a major factor influencing osteoporosis rather than the presence of diabetes. One of our patients (patient 3) had diabetes for only 1 year but had severe manifestations of KSS. Multiple comorbidities may have led to osteoporosis in that patient, and the direct effect of diabetes on skeletal health may be minimal given the very short duration of diabetes. In a study of 80 young patients with mitochondrial diseases, 14% had a history of fractures and the mean age of fracture was 6.4 years.^(^ ^10^ ^)^ The presence of at least one risk factor affecting skeletal health was observed in 70% of the cohort, and 30% had four or more risk factors for poor bone health.^(^ ^10^ ^)^ This finding suggests the severity of the primary disease in the presence of multiple comorbidities are implicated in bone fragility in this population. In our cohort, the presence of comorbidities such as renal impairment, stroke, low BMI, and muscle weakness were more prevalent in patients who were diagnosed with osteoporosis than people without osteoporosis.

Given the higher prevalence of hearing impairment, especially among those with m.3243A > G pathogenic variants, ophthalmological manifestations such as ptosis, external ophthalmoplegia, ataxia and peripheral neuropathy may all contribute to risk of falls and hence fractures in this population. We are not aware of any publication on fall risk and contributions of falls in fractures in people with mitochondrial diseases.

Elevated lactate level is one of the common features of mitochondrial disorders.^(^ ^4^ ^)^ Acidosis is known to promote bone loss, and elevated lactate‐induced acidosis may be one of the factors for poor skeletal health in people with mitochondrial diseases.^(^ ^18^ ^)^

Regarding the treatment of osteoporosis, two of our patients were treated with bisphosphonate and two patients were treated with anabolic agents. It is possible that fracture risk is higher than estimated based on areal BMD (aBMD) by DXA in these patients given multiple risk factors; current guidelines use similar criteria for defining osteoporosis in people with mitochondrial diseases as in the general population.^(^ ^10^ ^)^ We did not find any literature on the efficacy and safety of bisphosphonate versus anabolic agents in patients with mitochondrial diabetes.

Based on our own experience of managing people with mitochondrial diabetes and a review of the literature, we recommend that all patients with mitochondrial diabetes should have a DXA at their first visit as diabetes is correlated with severity of the disease. Clinicians should focus on nutritional counseling and physical activities to promote optimal weight and musculoskeletal health. Moreover, it is important for physicians to consider mitochondrial diseases in patients with type 1 and type 2 diabetes who have atypical features such as a young age of diabetes onset, low body weight, presence of sensory neural hearing loss, and osteoporosis or fractures at a young age.

Generalization of our research findings and discussion are limited by small sample size and retrospective study design. Diagnosis of osteoporosis was based on clinical diagnosis rather than systematic DXA BMD estimation. In conclusion, the prevalence of osteoporosis is higher among people with mitochondrial diabetes. Bone fragility in people with mitochondrial diabetes is multifactorial, including presence of diabetes, severity of disease, low BMI, and multiple comorbidities. Future research is needed to understand skeletal effects of mitochondrial diseases and prevention and treatment of bone fragility.

## Disclosures

VNS received research support through University of Colorado from NovoNordisk, Insulet, and Tandem Diabetes Care, and received honoraria through University of Colorado from Lifescan for advisory board attendance and from Dexcom, Embecta, and Insulet for speaking arrangements. AL is a consultant for Illumina and UCB and is a site PI for clinical trials sponsored by Travere, Stealth BioTherapeutics, and UCB.

## Author Contributions

**Kagan Ege Karakus:** Data curation; formal analysis; methodology; writing – original draft; writing – review and editing. **Varun Suryadevara:** Data curation; methodology; writing – review and editing. **Austin Larson:** Methodology; supervision; writing – review and editing. **Prathosh Gangadhar:** Conceptualization; data curation; methodology; writing – original draft; writing – review and editing. **Viral N Shah:** Conceptualization, data collection, curation, analysis, review and editing.

### Peer Review

The peer review history for this article is available at https://www.webofscience.com/api/gateway/wos/peer-review/10.1002/jbm4.10824.

## Supporting information


**DATA S1:** Supplementary information.
