# Supplementary material for: Skeletal Health in Patients With Mitochondrial Diabetes: Case Series and Review of Literature
Source: JBMR Plus. 2023 Sep 26;7(11):e10824. doi: 10.1002/jbm4.10824 (PMC10652178; doi:10.1002/jbm4.10824)
Supplement: Supplementary file 1 — DATA S1: Supplementary information. [file JBM4-7-e10824-s001.docx]

Supplementary Figure 1: Flow diagram of the literature search for the study

Initial PubMed search with different Boolean operators- 255 manuscripts

Excluded

Reviews- 65

Not relevant- 159

Studies in non-diabetic population- 2

Studies in patients with T1D and T2D only- 25

Manuscripts that had studied BMD/fracture in MD patients- 4

BMD- bone mineral density, MD- mitochondrial diabetes, T1D- type 1 diabetes mellitus, T2D- type 2 diabetes mellitus
